# Comparison of toxic effects of lead and copper and protective power of glutathione on oxidative stress parameters

**DOI:** 10.1192/j.eurpsy.2022.1825

**Published:** 2022-09-01

**Authors:** J. Jovanovic Mirkovic, G. Kocic, C. Alexopoulos, Z. Jurinjak

**Affiliations:** 1 The Academy of Applied Preschool Teaching and Health Studies, Biochemistry, Cuprija, Serbia; 2 Faculty of Medicine, Biochemistry, Nis, Serbia; 3 The Academy of Applied Preschool Teaching and Health Studies, Cuprija, Serbia, Medicine, Cuprija, Serbia

**Keywords:** Lead, Copper, DNase, Glutathione

## Abstract

**Introduction:**

Lead as an industrial pollutant can be detected at all stages of the working and living environment. Lead, based on its properties, solubility and mobility, accumulates in the soil, so that the average concentrations of oil in the soil are between 15 and 25 mg/kg (Radojević at all., 1999). Due to increased human activity, the amount of copper in the air, soil and water has increased. Glutathione (GSH) is an essential cofactor of many enzymes, such as: formaldehyde dehydrogenase, glyoxalase, prostaglandin endoperoxide isomerase, dehydrochlorinase and others. GSH is a biological redox in the metabolism of erythrocytes, it also plays a role in the transport of amino acids. Reactive forms of oxygen cause oxidative biomolecules (lipids, proteins, DNA) (Freidovich, 1999; Massaad i Klann, 2010).

**Objectives:**

The aim of this research was to examine the protective role of supplements GSH in conditions of chronic intoxication with sublethal doses of lead acetate and copper II sulfate.

**Methods:**

The preparation of biomaterials for testing and making homogenates of brain tissue of albino rats of Wistar strain was performed and the activity of acid and alkaline DNase was measured spectrophotometrically (Kocić i sar., 1998).

**Results:**

Lead otherwise “as soft Lewis acid” has a pronounced affinity for interaction with “soft bases” such as S-atoms of the thiol group in antioxidants, natural biomolecules and supplements in this case in glutathione.

**Conclusions:**

It can be said that GSH is a desirable supplement and antioxidant in the detoxification of reactive oxygen species in rats exposed to lead poisoning.

**Disclosure:**

No significant relationships.

